# Variety-seeking, learning and performance

**DOI:** 10.1371/journal.pone.0247034

**Published:** 2021-03-08

**Authors:** Gino Cattani, Ji-hyun Kim

**Affiliations:** 1 Department of Management and Organizations, Stern School of Business – NYU, New York, New York, United States of America; 2 Yonsei School of Business, Yonsei University, Seoul, Korea; University of York, UNITED KINGDOM

## Abstract

According to the *variance hypothesis*, variety-seeking or exploration is a critical condition for improving learning and performance over time. Extant computational learning models support this hypothesis by showing how individuals who are exposed to diverse knowledge sources are more likely to find superior solutions to a particular problem. Yet this research provides no precise guidelines about how broadly individuals should search. Our goal in this paper is to elucidate the conditions under which variety-seeking in organizations is beneficial. To this end, we developed a computational model in which individuals learn as they interact with other individuals, and update their knowledge as a result of this interaction. The model reveals how the type of learning environment (performance landscape) in which the learning dynamic unfolds determines when the benefits of variety-seeking outweigh the costs. Variety-seeking is performance-enhancing only when the knowledge of the chosen learning targets (i.e., individuals to learn from) provide useful information about the features of the performance landscape. The results further suggest that superior knowledge might be available locally, i.e., in the proximity of an individual’s current location. We also identify the point beyond which variety-seeking causes a sharp performance decline and show how this point depends on the type of landscape in which the learning dynamic unfolds and the degree of specialization of individual knowledge. The presence of this critical point explains why exploration becomes very costly. The implications of our findings for establishing the boundaries of exploration are discussed.

## Introduction

According to the *variance hypothesis*, variety-seeking or exploration is a critical condition for improving learning and performance over time [1]. Extant computational learning models support this hypothesis by showing how individuals who are exposed to diverse knowledge sources are more likely to find superior solutions to a particular problem [2–5]. Yet this research provides no precise guidelines about how broadly individuals should search. Although variety-seeking allows individuals to gain access to new information and knowledge [6–8], there are also diminishing returns to exploration [9]. In organizations, for instance, exploration that spans organizational boundaries often comes at the expense of the “time needed to understand how external knowledge can be integrated with the firm” [1, pp. 283]. As individuals expand their search, the amount of “new knowledge to be integrated into a firm’s knowledge base increases [and] so do the technological and organizational challenges in integration” [10, pp. 1185].

Previous research has discussed extensively how “learning and change are most often incremental and involve trade-offs between local expediency and more distant optimality” [11, pp. 356]. Yet much less attention has been devoted to establishing how broad exploration should be. Unless individual actors are assumed to have a more accurate mental representation of the performance landscape–i.e., the relationship between choices and performances [5, 12, 13]–the question of *where* in this landscape the requisite variety in terms of knowledge can be found has remained unattended. Our goal in this paper is to elucidate the conditions under which variety-seeking is more or less beneficial. We are therefore less concerned with the optimal balance between exploitation and exploration, which has been center stage in the learning literature, but we instead probe the boundaries of exploratory search by examining how broadly individuals can effectively search in different learning environments.

Following the lead of previous computational studies [2, 4, 14], we model learning as a process through which individuals interact with other individuals and, in the process, update their knowledge about the performance landscape. This interpersonal learning dynamic leads to novel combinations of knowledge, some of which represent superior solutions. This type of knowledge re-combination is common in business practices and often times may have a significant impact on organizational performance. For instance, the task of making of a new animation movie at Pixar is assigned to a group of individuals working very closely together for months. To enhance learning among group members, a well-established practice at Pixar is the daily review process of giving and getting feedback in a positive way–a practice called ‘the dailies.’ As Ed Catmull, co-founder of Pixar and former president of Walt Disney Animation Studios, put it: “People show work in an incomplete state to the whole animation crew, and although the director makes decisions, everyone is encouraged to comment” [15, pp. 7–8]. A key benefit of this practice is that people learn from and inspire each other by identifying and solving problems early in the production process instead of waiting for the movie to be completed, when making any change would prove extremely costly.

The results of the simulation reveal how variety-seeking does not *necessarily* lead to superior learning outcomes. The type of learning environment (i.e., performance landscape) in which interpersonal learning unfolds is critical for establishing when variety-seeking is beneficial. Our findings suggest that broadening search is performance-enhancing only when the knowledge of the chosen learning targets (i.e., individuals to learn from) provide useful information about the actual features of the performance landscape. The performance landscape can be single- or multi-peak. In the model, the number of peaks captures the extent to which interpersonal learning is frictionless, namely it is costless for individuals to interact and learn from each other; or some frictions make it costly to learn from other individuals who hold superior knowledge. Consider the case of Four Seasons–the luxury hotel chain. As David Crowl–former vice president of sales and marketing–pointed out, Four Seasons typically learns from each of its properties around the world because “we are an international hotel company, we take our learning across borders. At our new property in Egypt, we are going to try to incorporate indigenous elements to the spa, but we will still be influenced by the best practices we have identified at our two spas in Bali” [16, pp. 3]. Despite Four Seasons’ longstanding strategy of tailoring its properties to each location, the company encourages its employees to access the requisite variety available in other locations, so fostering interpersonal learning even across geographically distant properties, with people regularly visiting other locations to learn from or educate others. Yet, while some best practices (or superior solutions) can be learned and transferred across different locations, others are location-specific, i.e., valuable for one location but of little value or even harmful to another. In this case, the performance landscape can be modeled as a multi-peak landscape in which variety-seeking might prove very costly due to the frictions that make the sharing of superior knowledge less viable.

The results further reveal that the requisite variety can very often be available in the proximity of an individual actor’s current location in the performance landscape. For instance, when Apple approached Corning looking for an ultrathin glass for cell phones that could be touchstone but without breaking in case it fell, Corning had to decide whether to develop this type of glass from scratch or instead leverage its existing knowledge base. To develop such a glass, eventually known as the Gorilla glass, the team in charge resurrected Chemcor–a strengthened glass developed in the 1960s –which Corning had unsuccessfully attempted to apply in producing safety eyewear and windshields for cars, and shelved in September 1971 [11, 17]. In both cases, local as opposed to distant exploration proved to be a more effective search strategy. If the team had attempted to develop a completely new glass, it would never have succeeded in delivering it within six months, as Apple had requested, thus missing out on a very lucrative business opportunity. Unlike previous research findings, this implies that broad search may not be the most effective search strategy.

We further identify the point beyond which variety-seeking causes a sharp performance decline and show how this point depends on the type of landscape in which individuals learn from each other and their degree of specialization. The presence of this critical point explains why, as individuals change the scope of their search strategy, exploration becomes very costly. Extant research emphasizes the benefits accruing to distant search; yet viable opportunities might be available locally, that is, in the proximity of an individual’s current location. As our findings suggest, undertaking a ‘long jump’ [18] (i.e., distant exploration) may not be the most effective search strategy in this case. Therefore, we shed light on when distant search is useful in finding the requisite variety.

The paper is organized as follows. In the next section, we review extant computational research on learning and recent empirical studies that examine the impact of variety in knowledge on the effectiveness of search, i.e., the ability to identify superior solutions to a particular problem. We then explain the key features of our model and introduce the main questions that the model is meant to address. Next, we present the results of a series of experiments designed to analyze the influence of interpersonal learning on the effectiveness of search as individuals learn from other individuals whose knowledge is more or less similar to theirs under varying experimental conditions. The concluding section draws out the general implications of the simulation results and identifies model extensions that represent viable avenues for future research.

## Literature review

The most influential treatment of the variance hypothesis in organization studies can be traced to March’s [3] mutual learning model. In this model, organizational members learn from other members *indirectly* via an organizational code. The organizational code draws upon the knowledge of the best-performing individuals, i.e., individuals whose performance is higher than the organizational code’s performance; individuals, in turn, learn from the code. It is through this mutual learning process between the code and individuals that superior knowledge is disseminated indirectly (i.e., via the code) across the entire organization. As individuals learn from the code, they become socialized into the organization’s knowledge. Since the code can learn only from individuals who deviate from it, preserving the requisite variety in knowledge is critical to support the exploration that “allows the knowledge found in the organizational code to improve” [3, pp. 76]. Conformity to the organizational code, “drives out interpersonal heterogeneity, which results in lower long-run performance than would be possible through slower conformity to the code” [4]. One way in which the requisite variety can be preserved is through personnel turnover, with newcomers bringing along new knowledge. More generally, exploration creates and preserves the variety necessary for the organization to sustain its learning–as well as performance–in the long term.

Extending March’s [3] original model, other scholars have modeled learning as a process in which individuals learn *directly* from each other [2, 4]. Indeed, much of the learning that “goes on within organizations occurs directly from person to person and is not limited to exchanges mediated by organizational codes” [4, pp. 711]. Individuals compare the performance of their own knowledge to the performance of other organizational members’ knowledge: if they discover that this knowledge could help them further improve their performance, they may decide to update their knowledge by incorporating aspects of the higher-performing knowledge set. As individuals update their knowledge, over time organizational performance will also improve. Whether the organizational code learns from the best-performing individuals (and individuals, in turn, from the code) or individuals learn from the best-performing individuals by interacting directly with them, in both cases organizational performance improves over time.

The previous computational learning studies strongly support the variance hypothesis by showing how variety in knowledge reduces the risk of being trapped on a suboptimal part of the peak (i.e., an inferior technology or market product). By conducting exploration that spans both intra- [2, 19–21] and inter-organizational [6–8, 14] boundaries, individuals within an organization seek out the variety required to improve performance.

Two questions, however, remain unaddressed. First, this research assumes that no actual impediment other than structural inhibits the dissemination as well as the incorporation of superior knowledge. The emphasis on the performance-enhancing effects of variety in knowledge rests on the implicit assumption that there is a single, optimal set of knowledge that individuals incorporate by learning from other individuals within and across organizational boundaries [2–4]. Over time high performers’ knowledge is disseminated throughout the organization, thus contributing to improving its performance. The positive effect of incorporating such knowledge makes intuitive sense if the performance landscape in which individuals interact consists of a single, global peak–as the previous computational models implicitly assume. In this type of learning environment, there are knowledge complementarities that allow individuals to incorporate the knowledge of others, but without experiencing a performance decline. All individuals have to do is to update their knowledge by incorporating some of the better-performing individuals’ knowledge.

We provide numerical evidence on why the single-peak is qualitatively different from the multi-peak case in the “Probing the Model Assumptions” section (see below). When the assumption about a single, optimal set of knowledge is relaxed and knowledge is allowed to differ, the benefits of variety-seeking are less obvious because differences in individuals’ knowledge are costly to reconcile and integrate. This may occur because, for instance, inventors who are active in different technological domains often disagree about the type of technology or product to which a firm should allocate its scarce resources. Cognitive barriers may prevent them from working effectively together [22–24], personal agendas may inhibit the effective combinations of their knowledge, or simply knowledge cannot be reconciled, possibly resulting in combinations that prove inferior solutions to a particular problem. For instance, before the advent of digital imaging technology, Polaroid had adopted a ‘razor/blade’ business model. This model was so successful that the top mangement felt that Polaroid “could not make money on hardware, only software (i.e., film)” [25, p. 1152]. In the world of instant photography, this business model was effective because the learning environment was essentially single-peak. With digital imaging, the new learning environment was characterized by a new peak, whose attractiveness increased as digital cameras gradually replaced instant cameras. Polaroid created a division focused on digital imaging and a new set of knowledge emerged within the company; yet the razor/blade business model continued to guide key investment decisions, even though the model was less appropriate for digital cameras.

Extant learning models usually assume away possible frictions that constrain individuals’ ability to learn from each other. One the one end, exposure to a wider range of learning opportunities is expected to enhance performance; on the other, such exposure may prove very costly, therefore curtailing the expected benefits. This raises the question of how expansive exploration should be. In other words, what are the optimal boundaries of exploratory search?

To expose the tradeoffs that firms face in deciding how broad or focused their exploratory search should be, it is necessary to examine the learning dynamic unfolding in a multi-peak landscape where frictions and search boundaries can be explicitly modeled and analyzed. In previous models where individual actors are assumed to have the ability to determine the value of all alternative locations on the landscape [18], such frictions–in the form of search costs–do not affect learning outcomes (but for an exception see Rivkin [26]). To inspect when variety-seeking is a viable search strategy, the next section introduces a computational model in which individuals hold knowledge under conflicting realities. Part of this effort is to develop a simple, multi-peak landscape. Its relation to NK landscapes is also discussed in detail. We then probe the generalizability of the results by examining their sensitivity to varying experimental conditions.

## The model

Following previous computational research, we model an organization as consisting of a set of individuals located on a performance landscape. We start by considering the case of two distinct sets of individual knowledge: each set is a better fit for a different peak on the landscape. This means that the landscape has two peaks. Individuals located in the vicinity of the same peak are likely to be more similar as they share common knowledge about the region of the landscape in which they are located. The opposite holds for individuals located on different peaks. Returning to our Four Seasons example, this fits the case of individuals working in two hotels highly adapted to the local needs. To the extent that knowledge reflects local needs that differ substantially between locations, the benefits from interpersonal learning may diminish. For instance, Four Seasons hired a celebrity chef for the restaurant of its property in Paris for the first time in its history: this was an essential feature for a luxury hotel in a location where a Michelin-starred restaurant was expected. This ‘local’ knowledge would not be useful in other locations (especially in some Asian countries) where the possibility of choosing from among different dining options is expected. In such places, learning from the hugely successful experience in Paris would have proven of little use, if not downright harmful [16].

Individuals try to learn by incorporating others’ knowledge; whether or not this interaction enhances their fitness or performance level is represented by having individuals move up or down the peak on which they are located. We show how our main arguments and findings generalize to the case with (1) two peaks of equal height, (2) two peaks of unequal height, and (3) multiple peaks. The second case refers to when one location offers better learning opportunities, while the third case to when the reality is more complex and finding superior knowledge is increasingly difficult.

### Model of interpersonal learning on a two-peak landscape

Our model consists of three main parts: an external reality–which defines the shape of the performance landscape–individuals, and learning rules. In March [3] and other studies in the same tradition, an external reality is modeled as an *m*-dimensional vector where each dimension is assigned a binary value with equal probability. As a result, the randomly created reality corresponds to a landscape with a single peak. An alternative approach is to model the external reality as a multi-peak landscape that allows for the co-existence of different–including conflicting–realities (e.g., competing technologies or production methods, different markets or industries) to which individuals try to adapt.

**Fig 1:**
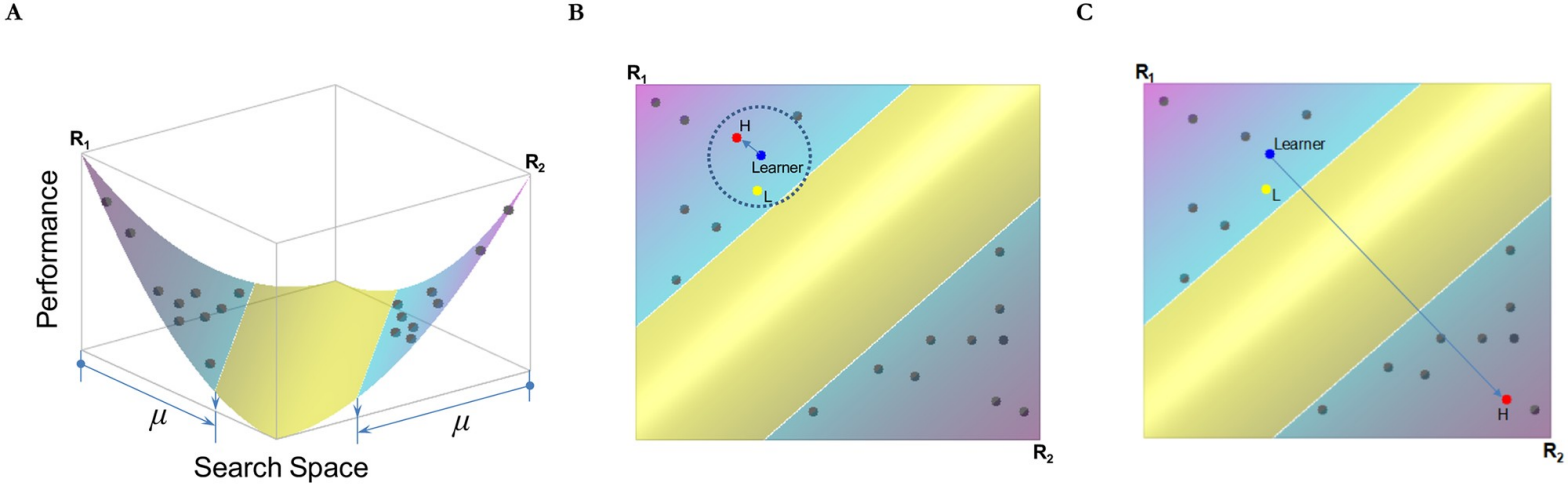
Learning on a two-peaked landscape with limited learning targets. **(A)** Performance landscape indicating there are two knowledge sets at two endpoints denoted by R_1_ and R_2_. **(B)** A contour plot view illustrating how learning unfolds under focused exploration (i.e., a small *σ* value; *σ* indicates search boundary). H and L indicates a high and a low performer within *σ*. **(C)** A contour point view in the case of broad exploration.

In the simulation, we start with the simple case in which the external reality coincides with a two-peak landscape. This is represented in Fig 1, an intuitive visual representation which assumes a U-shaped landscape, with each peak located at each end of the performance landscape [27, p. 189], for a two-dimensional representation. This implies that the two peak points correspond to two distinct realities sharing the least number of identical dimensions. We created two realities as *R*_1_ and *R*_2_. Specifically, *R*_1_ is denoted as (*r*_1_, *r*_2_,.., *r*_*i*_,.., *r*_m_) and *R*_2_ as (*r’*_1_, *r’*_2_,.., *r’*_*i*_,.., *r’*_m_)–where if *r*_*i*_ = 0, then *r’*_*i*_ = 1 and vice versa. We set *R*_1_ and *R*_2_ as (0, 0, …, 0) and (1, 1, …, 1), respectively. But one can use other values for *R*_1_ and *R*_2_ as long as *r*_*i*_ ≠ *r’*_*i*_. An individual’s knowledge about the external reality, denoted as *X* = (*x*_1_, *x*_2_,.., *x*_*i*_,.., *x*_m_), is also modeled as an *m*-dimensional vector with a binary value 0 or 1. An individual’s task is to match a reality as closely as possible. Thus, each dimension in the knowledge vector can be treated as a sub-task an individual has to work on. To create a U-shaped landscape, the performance of an individual, denoted as *Y*(*X*), is determined by the maximum value of the two functions that capture how closely an individual knowledge matches a specific reality. Following previous computational models, the height of a point in a landscape captures the level of performance corresponding to that point. For instance, a salesman is assigned the task of increasing the sales of a particular product on the market: the height of the peak indicates how well or poorly s/he fares in pursuing that goal. More formally, an individual’s performance is represented as follows:

$$Y(X)=f(R_{1},R_{2,}X)=max(\frac{1}{m}\sum_{i=1}^{m}\delta_{i,1},\frac{1}{m}\sum_{i=1}^{m}\delta_{i,2})$$ (1)

where *δ*_*i*,*j*_ = 1 if the *i*^th^ element of *X* matches the same dimension in the *j*^th^ reality and *δ*_*i*,*j*_ = 0 otherwise (note that *i*∈{1, 2, …, *m*} and *j*∈{1, 2}). Intuitively, the payoff function takes the maximum value of two payoff functions, each of which is an argument in the max function in (1). As the two extreme points are the peak points and the payoff decreases as we move farther away from each of them, taking the maximum value of the two superimposed functions results in a U-shaped payoff function.

We initially seed the same number of individuals on each peak. Let *n* denote the entire number of individuals in the organization. *n*/2 individuals are located on the *R*_1_ side while the rest are on the *R*_2_ side. The parameter *μ*∈(0,0.5] (i.e., 0 is not included in the set) captures how dispersed individual knowledge is at the beginning. Intuitively, *μ* indicates the range within which individual knowledge are initially located. Since the range starts from each end of the landscape, *μ* = 0.5, the maximum value of *μ*, implies that individual knowledge is dispersed over the entire range of the landscape. More formally, each element in an individual knowledge set *X*_*j*_ = (*x*_1,*j*_, *x*_2,*j*_, …, *x*_m,*j*_) on the *j*^th^ side follows the Bernoulli distribution *B*(1, U_*j*_), where U_*j*_ is uniform in [0, *μ*]. Each element on the other side, *X*_¬*j*_ = (*x*_1, ¬*j*_, *x*_2, ¬*j*_, …, *x*_m, ¬*j*_), follows the Bernoulli distribution *B*(1, U_¬*j*_), where U_¬*j*_ is uniform in [1- *μ*, 1]. For each individual, U_*j*_ or U_¬*j*_ is drawn independently.

Starting from each end point, most initial individual knowledge is distributed within the range represented by *μ*. As *μ* becomes larger and approaches 0.5, individuals are more spread out over the entire performance landscape and, therefore, the density of individuals located on a particular peak is lower. As a result, when *μ* is close to 0.5 individuals’ knowledge has not yet become specialized for any particular application (i.e., peak in the landscape).

In every period, all *n* individuals in the organization have the opportunity to learn from other organizational members: interpersonal learning is the mechanism through which individuals search for combinations of superior knowledge. The objective of learning is to adopt the attributes of the best-performing individual from a chosen sample of learning targets (see below). Individual performance is measured by Eq (1) and the best-performer is defined by an individual whose performance, *Y(X)*, is the highest within the chosen sample. Learning is ‘imperfect’ unless the sample of learning targets includes all the individuals in the organization. Each individual chooses *b* (1≤*b*≤*n*-1) individuals to learn from within a search boundary.

A search boundary, denoted by *σ*, is defined in terms of Hamming distance divided by *m* (total number of knowledge dimensions). The Hamming distance indicates the number of dimensions at which the corresponding values are different. Thus, the theoretical range of *σ* is 0 to 1. If the Hamming distance between the learner and another individual is smaller than *σ*, this individual is a potential learning target for the learner. When *σ* = 0, individuals do not learn from other individuals; when *σ* = 1, individuals potentially can learn from any other individual. Within the *σ* range, the learner randomly chooses *b* individuals and then learns from the best-performing individual by copying a *p* fraction of the attributes of the learning target (i.e., the best performing in the sample). Since only a fraction of the attributes of the best performing individual in the sample is copied, learning is always imperfect. This model feature is intended to enhance the empirical plausibility of our model: some knowledge is difficult to absorb due to, for example, its tacit nature and therefore cannot be easily communicated even when individuals are otherwise willing to share it. Indeed, the parameter *p* is meant to capture the fact that even if one can identify the best performing individual in a group or organization, only a fraction of this individual’s knowledge can be absorbed. Our model does not distinguish whether this is due to the difficulty of communicating certain knowledge dimensions or the reluctance to share them with others. Yet the ability to identify the best performing individual is possible in several situations. Since 1972, for instance, the *Institutional Investor* magazine has annually ranked the top analysts in equity industry group and investment specialty using four levels of awards: first place, second place, third place, and runner-up [28]; the number of awards and nominations individuals receive or the commercial success of their work is often used to recognize top-performers in industries like film-making, music, advertising, TV and publishing, among the others [29]; and scientists and engineers who file the largest number of patents and/or publish articles in prestigious scientific journals are regarded as top-performing employees in many R&D intensive firms. For example, IBM has its “IBM Fellows” award to celebrate the achievements of star performers and 3M honors its top scientists through their induction into the Carlton Society [30]. It is therefore possible to identify best-performing individuals based on a series of key performance indicators that are available and widely used within and outside organizations operating in the same industry.

While individuals can identify the best-performing individual among the chosen ones, they cannot anticipate the outcome quality of their learning efforts. In other words, individuals have no foresight about the learning outcome. For instance, prior research [28, 29] has shown how teams staffed with star performers only perform well after their members (i.e., stars and non-star performers) have learned to work together over time: since star performers tend to have big egos, they usually expect their co-workers to adapt to their work style. This is why some firms that consider hiring star performers often hire their close collaborators in order to recreate the same basic work environment that allowed stars to reach the level of performance they had while working for their former employer. In this sense, our model differs from previous models that assume individuals to have such ability [13, 18, 31, 32]. This assumption implies that no performance decline is observed when individuals learn from each other. We discuss this important point in the section “Relationship with Prior Models” (see below). The results that incorporate foresight and errors in evaluation are also described in the sensitivity analysis section.

Fig 1 offers an intuitive visual representation of the interpersonal learning dynamic over a two-peak landscape. In Fig 1A, the U-shaped performance landscape indicates that there are two knowledge sets at two endpoints denoted by R_1_ and R_2_. We also tested the model on a bimodal landscape in which the peak points were located in an interior part of the landscape: the results were qualitatively similar to those of our baseline model. The range is colored on the landscape surface with a different color: pink and blue for the range *μ* and yellow for the rest (in a black and white color scheme, the *μ* range starts from each end of the landscape and stops where there is a change in the color scheme). When *μ* is small, individuals located in the vicinity of the same peak tend to be more similar to each other because they share similar knowledge.

The degree of search boundary determines the extent to which the knowledge that individuals incorporate by interacting with one another are diverse. Fig 1B, a contour plot view of Fig 1A, illustrates how learning unfolds under focused exploration (i.e., a small *σ* value). Each individual seeks a sample of other individuals to learn from within a certain range. The range of this search is expressed as the radius of the circle surrounding the ‘Learner.’ The radius thus represents *σ*: The larger the radius, the larger the value of *σ*. As we described earlier, in the actual computational model, *σ* is defined in terms of Hamming distance divided by *m*. This implies that, if the Hamming distance divided by *m* between the learner and another individual is smaller than *σ*, the individual is a potential learning target for the learner.

Fig 1B also illustrates how the ‘Learner’ randomly chooses *b* individuals within the *σ* range (in the current example *b* = 2 and, therefore, there are two individuals within the search boundary). Among the chosen *b* individuals (denoted by red and yellow dots in a colored version), the learner learns from the best-performing individual. Accordingly, in the current example, the individual denoted by *H* (for High Performance) is chosen as a learning target and the learner adopts *p* fraction of the learning target’s knowledge. On the landscape figure, we can express this learning dynamic by moving the learner closer to the individual marked by *H*. Fig 1C illustrates the case with broad exploration (i.e., *σ* = 1). Due to the broad nature of the search boundary, the learner can choose an individual who is located on the other peak. In the current example, the individual on the other peak exhibits a higher performance and so is chosen as a learning target.

### Relationship with prior models

Our model differs from the NK modeling approach in several respects. The main difference is the nature of landscapes. As the generation of landscapes in the NK models relies on a random number generation process, knowing precisely how any particular choice configuration interacts with other choice configurations is difficult to fathom. Consider the following example. A configuration of choices with all 0’s would have a different fitness level from a configuration with all 1’s (unless two random draws from the uniform distribution corresponding to contribution values for the 0-element and 1-element happen to have the same value). This is the case regardless of the values of N and K. Let’s assume that N = 3 and K = 1.

To calculate the performance value of 000 and 111, the only contribution values we need to know are for 00 and 11 because each choice shares an interdependence relationship with a subsequent choice (e.g., the contribution of the first 0 in 000 is influenced by the second 0). Let’s further assume that, for example, the random draw performance contribution for 00 is 0.53 and for 11 is 0.75 (or you can draw any two numbers randomly between 0 and 1). Then, fitness levels for 000 and 111 are 0.53 and 0.75, respectively. Because fitness calculations for other values between 000 and 111 –e.g., 010 –require additional random draws, recombining 000 and 111 involves uncertainty about whether the resulting choice configuration will have inferior or superior fitness levels. In the NK setting it is not clear whether there is a conflicting relationship between 000 and 111, nor does the modeler have control over that relationship. In our model, on the contrary, we can impose a conflicting relationship between two choice configurations according to the modeler’s intentions. In the S1 Appendix, we show how the results of the simulation when an NK modeling approach is used still support the variance hypothesis: on average broad exploration leads to higher performance or superior solutions to a given problem-solving situation without incurring costs.

The other main difference is the rule of adaptation. Our rule of adaptation assumes that agents are even more boundedly rational than in previous models where agents are assumed to have the ability to evaluate new choice configurations, even before implementing any changes, in both local and distant search [18, 32] (see also Ganco and Hoetker [33] for other search rules in the NK modeling setting). In the canonical NK models, agents can evaluate new alternatives, regardless of their locations. In the case of local search, “if the organization’s performance declines, then the organization returns to its prior starting point for its subsequent efforts at local search” [13, pp. 123]. In the case of a long-jump, “an organization draws at random a new organizational form in the space of 2^*N*^ alternatives. The organization then compares the fitness value of this new organizational form with its current form and adopts the new form if it is superior to its current one” [18, pp. 938]. By relaxing these assumptions, our approach is similar to Posen et al.’s [14] model of imperfect imitation: while their model looks at how firms learn from other firms via imitation, we argue that a very similar learning dynamic may unfold also at other levels of analysis.

In Posen et al.’s model, firms are modeled as a configuration of an *m*-dimensional vector which corresponds to an individual knowledge in our and March’s model [3]. Thus, to follow the ‘keep-it-simple’ principle and focus on inter-entity dynamics, it is common to model both a firm and an individual as an *m*-dimensional vector. Within the range of *μ*, the way individuals choose other learning targets is the same as the imitation rule in Posen et al. Although we model inter-personal learning behaviors, our model can also be used in other settings like when, for instance, learning occurs among individuals in different organizational settings. Besides the fact that we model inter-personal learning while Posen et al. modeled inter-firm imitation, it is important to note that there are two major differences. First, while our major theoretical focus is on search boundary, *μ*, there is no restriction in search boundary in Posen et al. Their major restriction in search is the number of firms one can consider as imitation targets. Thus, how far or closely an imitation target is located is not an important consideration. Second, we set the situation with conflicting constraints (i.e., multi-peak) as a baseline. The type of situation Posen et al. consider is a single-peak landscape: although they allow for interdependence among choices by letting one attribute be interdependent with *k* other attributes, what they model is essentially a single-peak landscape. There is a single set of attribute configuration that gives the highest performance (i.e., when all the firm level attributes coincide with the attributes of the external reality) and all other configurations are strictly inferior–and the performance gap with the best-performing attributes gets larger as the number of non-identical attributes between the firm level attributes and those of the external reality increases.

An important advantage of introducing a more conservative assumption on bounded rationality is the possibility of modeling the costs of combining diverse knowledge explicitly. In previous models, individuals are assumed to have the ability to evaluate new alternatives and so they do not bear such costs. Rivkin labeled these costs as “search costs” and assumed them away from his model: “search costs are set to zero; indeed, the incremental improver knows the value of all neighboring strategies within a radius of *M* perfectly without cost or commitment” [26, pp. 829]. In our model, we define cost as intertemporal performance decline. It is not a direct way of modeling cost involved in search, but an “ex-post” search cost. For instance, if performance decreases from time *t* to *t*+1, the organization incurs a cost. Since we consider the performance difference between two consecutive periods, a cost can be applied to any period except for the first period. Thus, we examine whether an organization incurs a cost in the short or long run, or both. It is possible to incorporate search cost more directly by subtracting a certain portion of the achieved performance level in proportion to the search distance. This would, in principle, reduce the reported performance level and the amount of decrease would be proportional to *μ*. What might be more interesting is to assume individuals who can do marginal calculations, seeking the point where the marginal cost is equal to the marginal benefit of search. Since this requires another level of rationality, we do not incorporate this possibility in the current study. We also report the results of a series of sensitivity analyses in which individuals have foresight about what would happen as a result of learning. We find that, unless individuals have very strong foresight, our main results still hold.

An additional point on the relationship with prior imitation and recombination models needs clarification. The “identifying the best performer in the population” imitation rule–i.e., Rivkin’s [26] “follow-the-leader” imitation strategy–is a special case of our interpersonal learning model. Once we increase *b* (learning target size) to its maximum level, the learner copies the traits of the best performer.

### Simulation

We analyze the effect of exploration boundary by varying the level of *σ*. A high level of *σ* (i.e., broad exploration) implies that individuals learn from other individuals who are located on the same or different peaks. In all results reported below, we computed the average of 100 simulated organizations. Unless otherwise specified, we used the following parameter setting. The performance landscape consists of 50 dimensions (*m* = 50), meaning that there are 50 independent tasks each individual needs to perform. Each organization consists of 100 individuals (*n* = 100), while the learning rate (*p*) and the learning target size (*b*) are set at 0.3 and 2, respectively. In the sensitivity analysis sections (see below), we vary these values and confirm that our baseline results are robust under different experimental conditions. Each simulated organization allows individuals to learn from one another until there is no better solution to be found–i.e., a steady-state.

Since we are concerned with the steady-state performance as well as the performance trajectory before reaching the steady-state, we explicitly account for the costs that are incurred to reach that state. Consistently with prior learning models, and in order to ensure comparability between the results of our model and those of these prior models, we measured the average performance across all individuals as a proxy of team or organization level performance. Depending on the number of individuals, the average performance can in fact represent performance at the team or group level, including the case of an organizational unit (e.g., a division) or a small firm (e.g., start-up) whereby it is plausible to assume that individuals not only know but also interact with one other. Thus, hereafter we call it performance. Table 1 reports all the parameter values used to generate the results below.

**Table 1:** Parameter values for presented results.

Parameter	Remarks	Range of parameter values in the presented results
*n*	Number of individuals	100
*m*	Number of dimensions in the external reality and individual knowledge	50
*μ*	Initial dispersion of individual knowledge	0.1, 0.5
*b*	Learning target size	2
*σ*	Search boundary in learning	0.3 (more restrictive search), 0.83, 1.0 (no restriction)
*p*	Learning rate (i.e., copying *p* fraction of the target attributes)	0.3

## Results

We now present the main results of our simulation model. We consider the impact of variety-seeking on performance over time under different exploration boundaries (parameterized by *σ*). The goal of the first experiment is to understand when incorporating different knowledge is more or less likely to be beneficial. In results not reported here, we also examined the role of exploration boundary when there is only a single set of optimal knowledge (i.e., the landscape has a single peak). Under this scenario, consistent with prior models [3], broader exploration (i.e., larger *σ*) provides access to greater knowledge variety and enhances performance without causing a performance decline. We further distinguish between the case in which the landscape has two peaks of equal height and the case in which the two peaks have uneven height.

### Learning in the presence of different optimal knowledge

**Fig 2:**
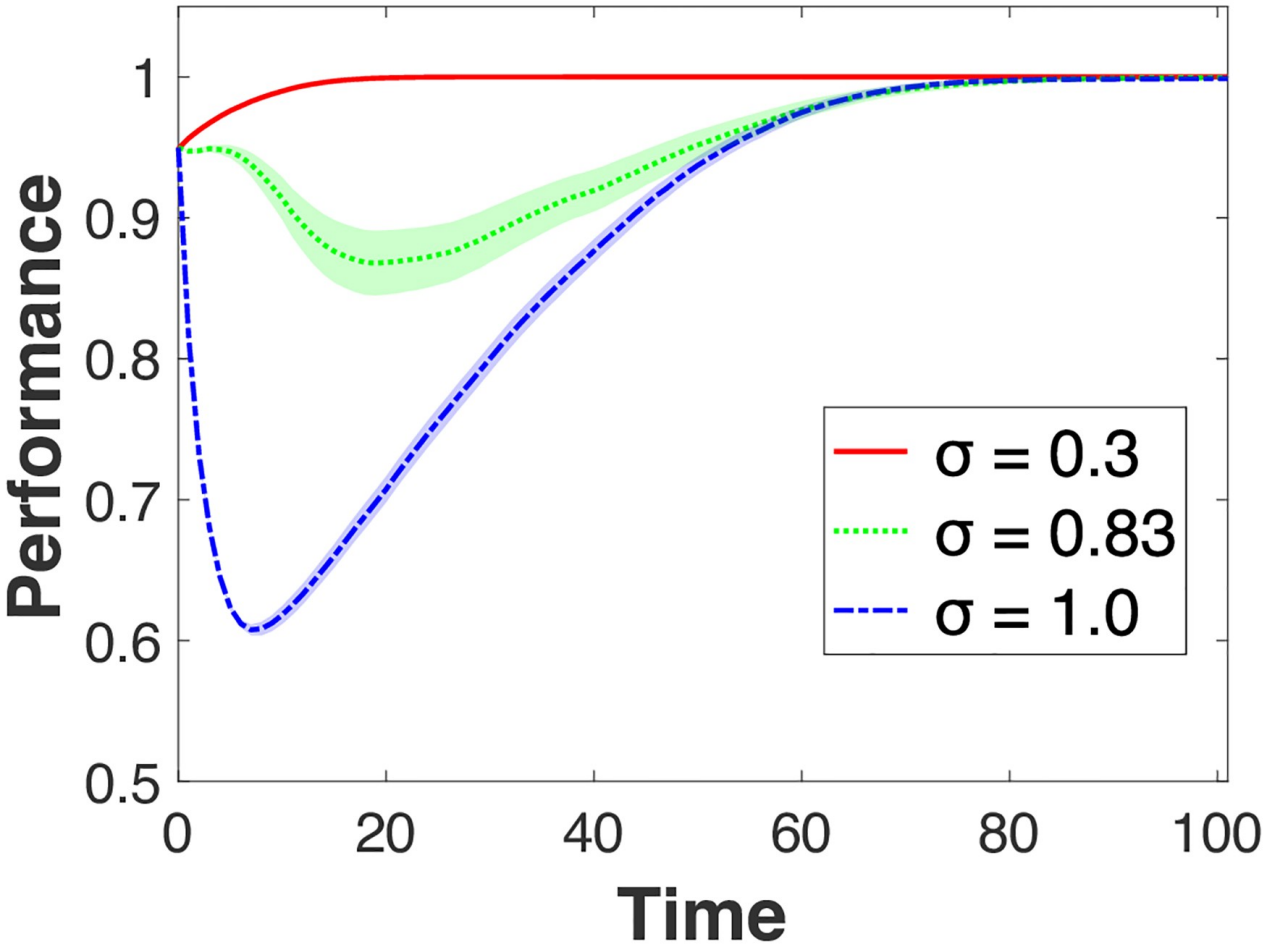
Effects of search restriction (*σ*): An equal peak case. The shades represent 95% confidence intervals. *μ* (initial dispersion of individuals) is set to be 0.1.

Fig 2 presents the average performance over time for different levels of *σ* with 95% confidence intervals. We fixed *μ* (initial dispersion of individual knowledge) at 0.1. For presentation purposes, we chose three levels of *σ* – 0.3, 0.83, and 1.0 –each representing a focused, moderate, and broad exploration scope for learning targets, respectively. We chose *σ* = 0.83 because it is a level of exploration that exhibits a performance pattern between the two extreme cases. In other words, a transition occurs at around *σ* = 0.83. Such transition emerges only within a small range of *σ* around 0.83. As we elaborate later, where the transition point occurs depends on the type of learning environment in which individuals interact, namely whether individuals’ knowledge is more or less specialized (as captured by *μ*). Thus, the transition range can vary depending on how specialized individuals’ knowledge is around two different peaks. We elaborate on the meaning of this range in the discussion. Decreasing or increasing *σ* above and beyond this range results in patterns similar to *σ* = 0.3 or *σ* = 1.0, respectively. Two findings are noteworthy from Fig 2: (1) there is a significant performance decline initially for a broad range of exploration (i.e., *σ* higher than 0.83); (2) the degree of variance in performance is largest for *σ* = 0.83 (i.e., the range for 95% confidence interval is widest for *σ* = 0.83). The maximum variance across all time periods for *σ* = 0.83 is 68.5 (when Time = 20), while it is 0.07 and 11.8 for *σ* = 0.3 and 1.0, respectively.

The first finding shows how interpersonal learning experiences a significant performance decline before reaching the maximum performance level. This decline is due to the cost that individuals incur as they try to learn from other individuals located on a different peak. When individuals maintain focused exploration (e.g., *σ* = 0.3), on the contrary, performance increases in a steady manner and eventually reaches the maximum level. When individuals engage in very broad exploration (e.g., *σ* = 1.0), interpersonal learning reaches the steady-state more slowly compared to the other two levels of *σ*, but also experiences a sharp performance downturn initially. This model thus reveals the costs of variety-seeking in learning that do not emerge in prior modeling studies.

Why do we observe such costs under the broad exploration condition? A rather intuitive explanation is that the incorporation of diverse knowledge is not automatic. As individual knowledge is ‘specialized’ for a given peak (i.e., useful for a specific task or application), it may be costly to incorporate the knowledge of individuals located on other peaks. As individuals do not know the actual shape of the landscape, they can experience a performance decline even if they learn from better-performing individuals located on a different peak. The Four Seasons example of hiring a celebrity chef at its property in Paris is an apt illustration of this particular situation, whereby knowledge that reflects too closely the reality of one location is much less valuable in a different location. Visually (Fig 1), this decline corresponds to an individual being repositioned in the middle area of the landscape.

In subsequent periods, underperforming individuals try to improve their performance by learning from better-performing others. In choosing the learning target, neither the location of the learning target nor the peak toward which the learner might move is known a priori. This implies that a learner who lands on the landscape’s middle area can move back and forth between the two peaks. Accordingly, the learner’s performance level will also fluctuate. In contrast, under focused exploration (*σ* = 0.3) individuals located on one peak would not consider individuals on the other peak as learning targets. Learning occurs among individuals *within* each peak, but not *between* peaks. Under focused exploration, therefore, the performance outcome resembles interpersonal learning on a single landscape: steady increase in performance without significant costs.

**Fig 3:**
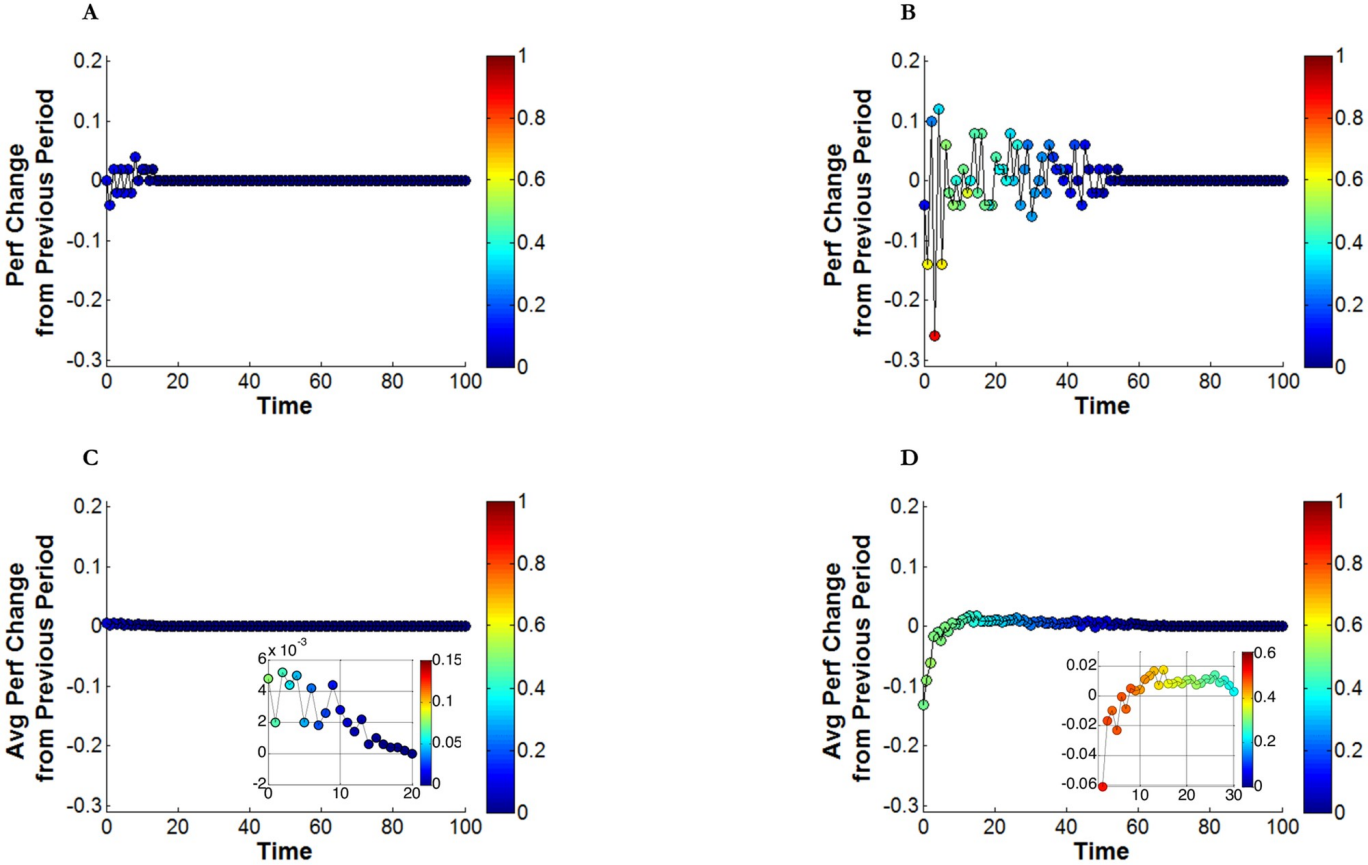
Search restriction and intertemporal variation in performance. Colors in data plot and color bars represent Hamming distance between the source and the learning target. **(A)** *σ* = 0.3 for a single Individual **(B)** *σ* = 1.0 for a single Individual **(C)** *σ* = 0.3 for average of 100 individuals **(D)** *σ* = 1.0 for average of 100 individuals.

To confirm our conjecture regarding the costs under broad exploration, we measured intertemporal performance variation between time *t* and *t*+1, and the Hamming distance between the source (i.e., the learning individual) and the learning target. A high degree of intertemporal performance variation would indicate that learners change their positions more frequently, moving back and forth between the two peaks, instead of steadily improving their performance by learning from other individuals located in the vicinity of the same peak. We thus expect performance variations over time to be larger for the high *σ* case, but smaller for the low *σ* case. We also expect that the Hamming distance between the learner and the learning target to be larger (i.e., the source learns from a target located at a more distant position) when the learner experiences a larger performance decline. The top panels in Fig 3 (Fig 3A and 3B) show intertemporal performance variations from a single realization for focused and broad exploration under the same parameters as in Fig 2.

When *σ* = 1.0 (Fig 3B), we observe two significant patterns compared to the low *σ* case. First, the degree of intertemporal performance variation is much larger. While the range of performance variation under *σ* = 1.0 is as large as ±0.2, it becomes as small as ±0.05 when *σ* = 0.3. Second, the length of the fluctuation is significantly longer. Fluctuation lasts until *t* = 55 under *σ* = 1.0, while it ends before *t* = 20 when *σ* = 0.3. We can thus conclude that individual learners experience a higher degree of performance fluctuation for a longer period when exploration is broader (*σ* = 1.0). The Hamming distance between the learning source and the target helps better understand the relationship between the learning target’s location and the resulting learning outcome. In Fig 3B, the learning events that resulted in inferior performance (below the zero point) were mostly from learning targets located in distant areas of the landscape (colors near red on the colorbar, i.e., close to the value 1 on the vertical bar on the right end side the figure). This pattern is confirmed in Fig 3A, in which learners choose targets nearby and so do not experience performance fluctuations.

The patterns from the previous two cases are observed not only in this particular single realization, but also in a more general setting. The bottom two panels in Fig 3 (Fig 3C and 3D) repeat the same experiment of the panels above, except that the results are based on averages across 100 individuals. The costs involved in learning from distant individuals become more apparent. In Fig 3D, for the first several consecutive periods, learners experience decreasing performance. In the subpanel of Fig 3D, we provide a magnified view from *t* = 3 to *t* = 30. From this, we can see that a performance decline during the first ten periods is followed by performance improvement. This pattern is consistent with, but also explains, the initial performance decline in the case of *σ* = 1.0 observed in Fig 2. On the other hand, the magnified view in Fig 3C explains the consistent performance increase in the case of *σ* = 0.3. By learning from nearby targets, individuals experience performance improvement for the first twenty periods, consistently with the performance-enhancing pattern in the case of *σ* = 0.3 (Fig 2). So, the comparison between the two *σ* cases suggests that learning from individuals whose knowledge is more diverse (i.e., *σ* = 1.0) entails a higher level of performance variation for a longer period compared to learning from individuals whose knowledge is less diverse (i.e., *σ* = 0.3). This is the source of initial costs that learners experience as they try to learn from individuals holding different knowledge.

The next question is why the degree of variance in performance is largest for *σ* = 0.83. From the first finding–performance decline resulting from incorporating diverse knowledge–and the explanation we gave in the previous paragraph, it follows that values of *σ* less than 1.0 should generate less variance in performance than the value of *σ* = 1.0. One might expect this to happen because the performance decline is the largest for *σ* = 1.0. However, we observe that variance in performance tends to be higher for *σ* = 0.83 than *σ* = 1.0. What is the reason for this unexpected result?

To answer this question, we need to examine performance variations across multiple simulation runs, namely beyond the intertemporal performance variation within a single simulation run. One way to do so is to determine whether all individuals converge to the top of the peak on which they were located initially or they all converge to the same peak, regardless of their initial location. It turns out that eventually every individual reaches a peak. Intuitively speaking, for small *σ* values, a certain proportion of individuals will converge toward one peak and the rest toward the other; for high *σ* values, all the individuals will converge toward one single peak, that is, the peak where the best performing individual is located. As we show below, a less intuitive pattern takes place for a small range of values of *σ* around 0.83 and this is what generates the high variance in performance.

**Fig 4:**
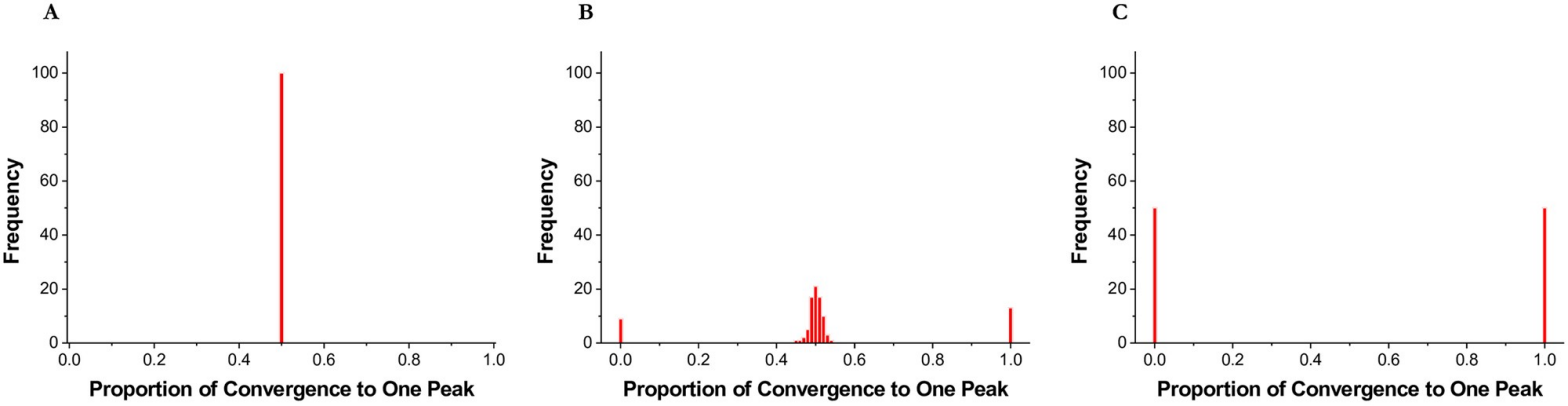
Distribution after convergence. On the horizontal axis, we indicate the proportion of individuals who converge to one peak (we picked the R_1_ peak.). On the vertical axis, we indicate the frequency of each proportion out of 100 independent runs. **(A)** *σ* = 0.3 **(B)** *σ* = 0.83 **(C)** *σ* = 1.0.

In Fig 4, we present varying converging patterns across different levels of *σ*. On the horizontal axis, we indicate the proportion of individuals who converge to one peak (we picked the R_1_ peak.). On the vertical axis, we indicate the frequency of each proportion out of 100 independent runs. The parameter setting is the same as in Fig 2. When *σ* = 0.3 (Fig 4A), all 100 cases have a proportion value of 0.5. This implies that in each of the 100 runs, one half of the individuals converges to the top of one peak (the R_1_ peak) and the other half to the top of the other peak (the R_2_ peak). Specifically, all individuals reach the top of the peak on which they were initially located. However, when *σ* = 1.0 (Fig 4C), a different pattern emerges. In 50 out of 100 cases, individuals converge to one peak; in the other 50 cases, individuals converge to the other peak. In each simulation run, all individuals converge to one peak with probability 0.5. The case with *σ* = 0.83 (Fig 4B) exhibits a pattern that lies in between the two extreme cases: 78 cases show a pattern similar to the case of *σ* = 0.3, while 22 cases are similar to the case of *σ* = 1.0.

Our results in Fig 4 indicate that when *σ* = 0.83, individuals may converge to one peak or spread out over two peaks. Predicting ex ante whether a single-peak or two-peak convergence is likely to ensue is not possible. Sometimes individuals converge to the peak closer to their starting position; other times individuals converge to a single peak after experiencing a significant initial performance decline. A combination of these two possibilities causes a high variance in performance.

**Fig 5:**
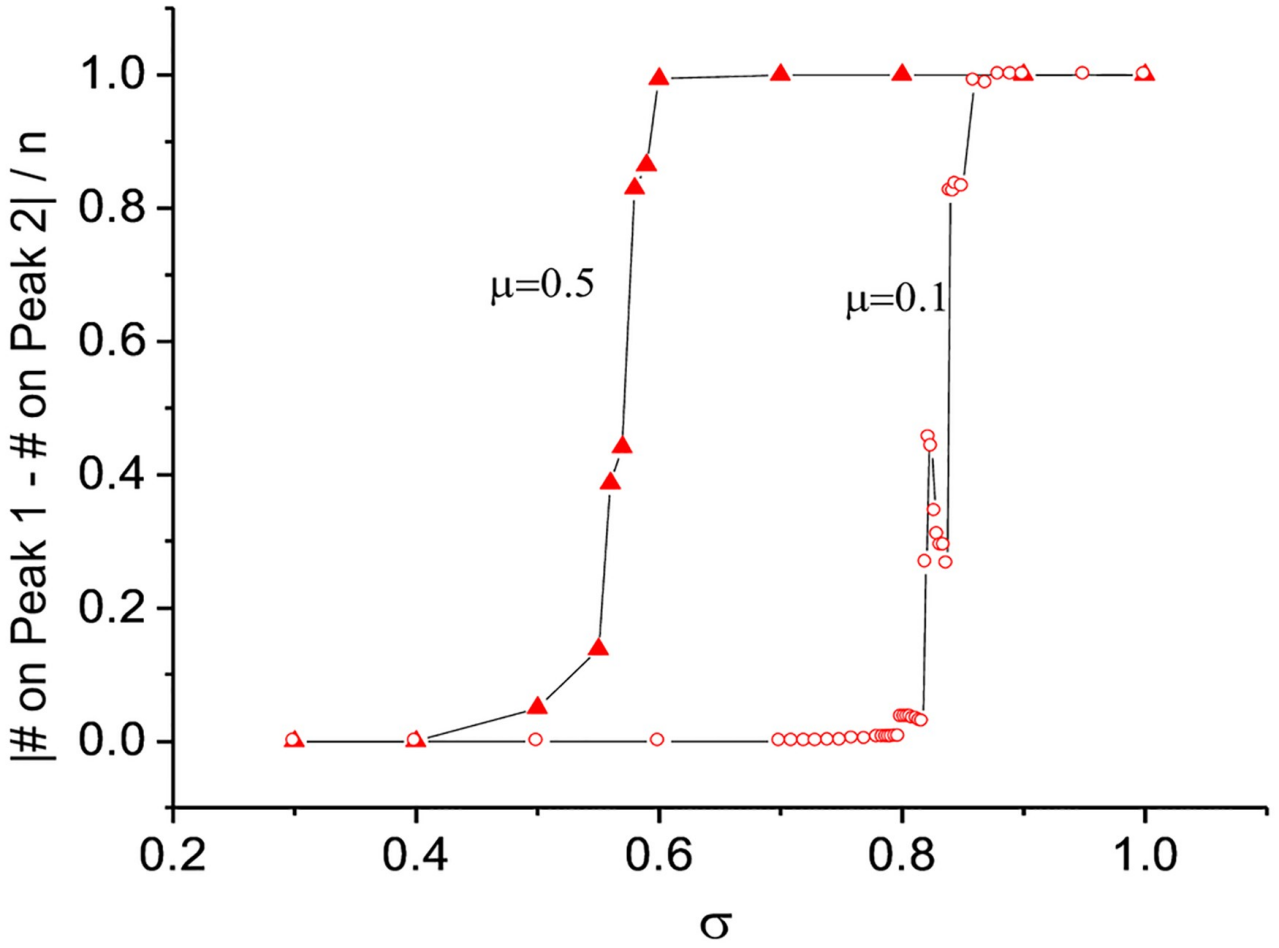
Transition to one-peak convergence. On the vertical axis, we report the absolute value of the difference between the number of individuals on one peak and the number of individuals on the other, divided by the entire number of individuals. If this ratio is 0, it means that the number of individuals on each peak is the same. For example, if 50 out of 100 individuals are on one peak and the other 50 are on the other peak, the value is |50–50|/100 = 0. As the ratio approaches 1, this means that there are more individuals on one peak than on the other. When the value is equal to 1 (i.e., |0–100|/100 = 1), all individuals are located only on one peak.

We now ask whether the non-deterministic convergence pattern takes place in a wide or narrow *σ* range. From the results above, it is apparent that a transition from two-peak convergence (i.e., individuals are spread out over two peaks) to one-peak convergence (i.e., all individuals converge to one peak) occurs near *σ* = 0.83. In Fig 5, we examine whether this transition happens abruptly or in a more gradual manner. Understanding how this transition unfolds is important because when the transition is abrupt, expanding exploration boundaries might prove costly. On the vertical axis of Fig 5, we report the absolute value of the difference between the number of individuals on one peak and the number of individuals on the other, divided by the total number of individuals. If this ratio is 0, it means that the number of individuals on each peak is the same. For example, if 50 out of 100 individuals are on one peak and the other 50 are on the other peak, the value is |50–50|/100 = 0. As the ratio approaches 1, this means that more individuals are on one peak than on the other. When the value is equal to 1 (i.e., |0–100|/100 = 1), all individuals are located on a single peak.

In Fig 5, we report the transition pattern for two values of *μ*. For *μ* = 0.1 the transition to a ‘one peak convergence’ takes place when *σ* = 0.83, whereas for *μ* = 0.5 the transition begins when *σ* is around 0.5. These differences in transition patterns suggest that the point at which the costs of variety-seeking in learning outweigh the benefits depends on whether individuals’ knowledge is more or less specialized (as captured by *μ*). Although the starting points differ, in both cases the non-deterministic convergence pattern occurs within a narrow range of *σ*. In principle, a transition from two-peak to one-peak convergence can be either continuous or discontinuous [34]. However, precisely classifying the current transition patterns into one of the two types requires more computational analyses and is beyond the scope of our study.

### High vs. low levels of knowledge specialization

**Fig 6:**
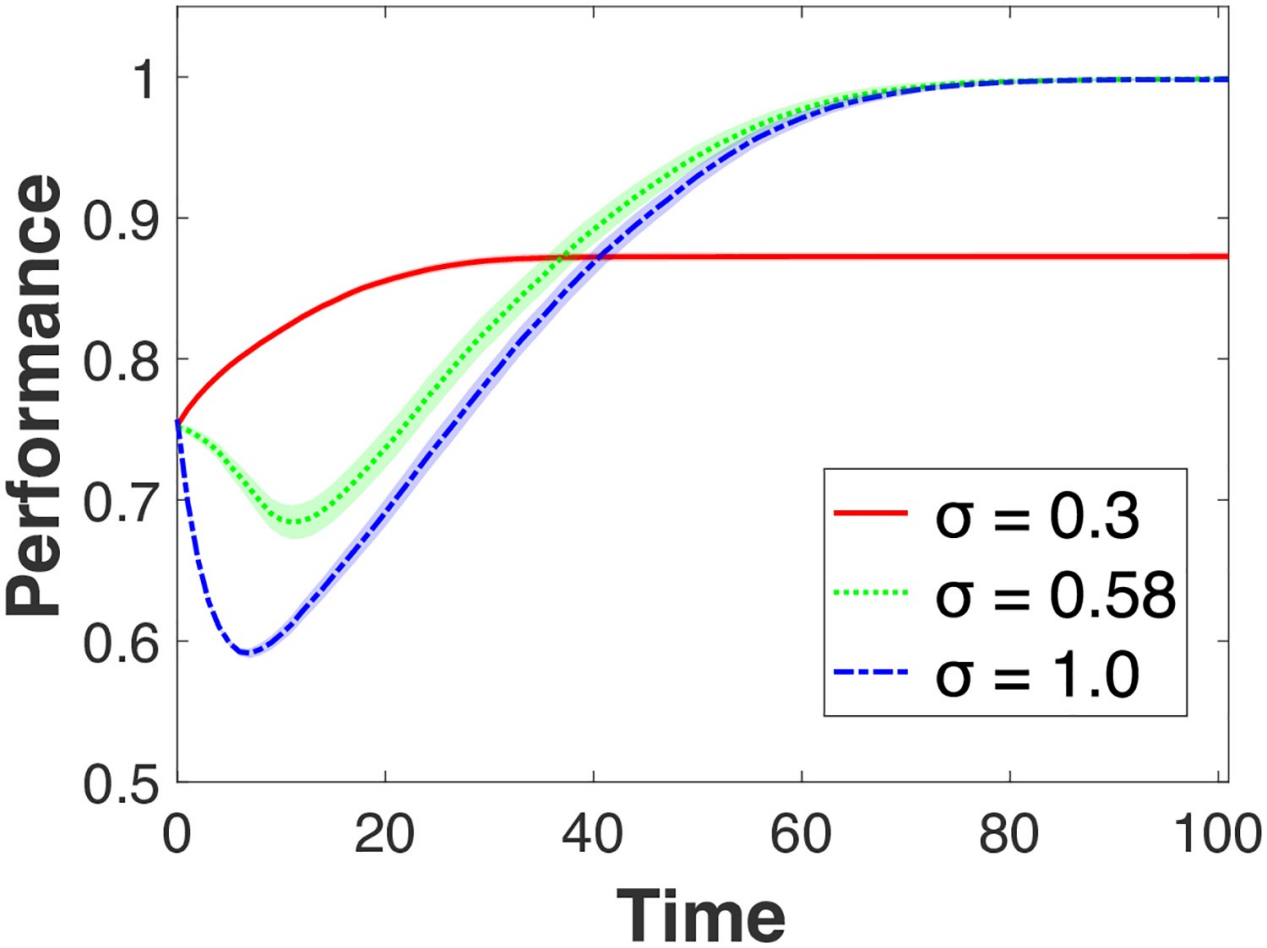
Effects under high initial dispersion of individual knowledge (*μ* = 0.5). The shades represent 95% confidence intervals.

We also examine the effect of different levels of knowledge specialization by varying *μ*, the initial diversity. Fig 6 shows the effect of *σ* when *μ* = 0.5 across different levels of *σ*. A high level of *μ*, implies that there are only a few ‘experts’ (i.e., individuals tend to be more spread out and located in the low/middle region of the landscape). Having more expert individuals seems to enhance learning.

This beneficial effect is represented by the initial performance levels in Figs 2 and 6. The initial level of performance for *μ* = 0.1 is much higher (around 0.95) than when *μ* = 0.5 (around 0.75). However, this benefit comes with a substantial cost. As in Fig 2, the degree to which performance decreases is much larger when *μ* = 0.1. While performance drops from 0.95 to 0.619 (the difference is 0.331) under *μ* = 0.1, it drops from 0.753 to 0.601 (the difference is 0.152) under *μ* = 0.5. When individuals are located close to the top of different peaks, their knowledge becomes more specialized. Hence, in this case, variety-seeking (i.e., incorporating the knowledge of individuals from a different peak) may dampen performance because this specialized knowledge cannot be effectively combined.

### Superior vs. inferior knowledge

**Fig 7:**
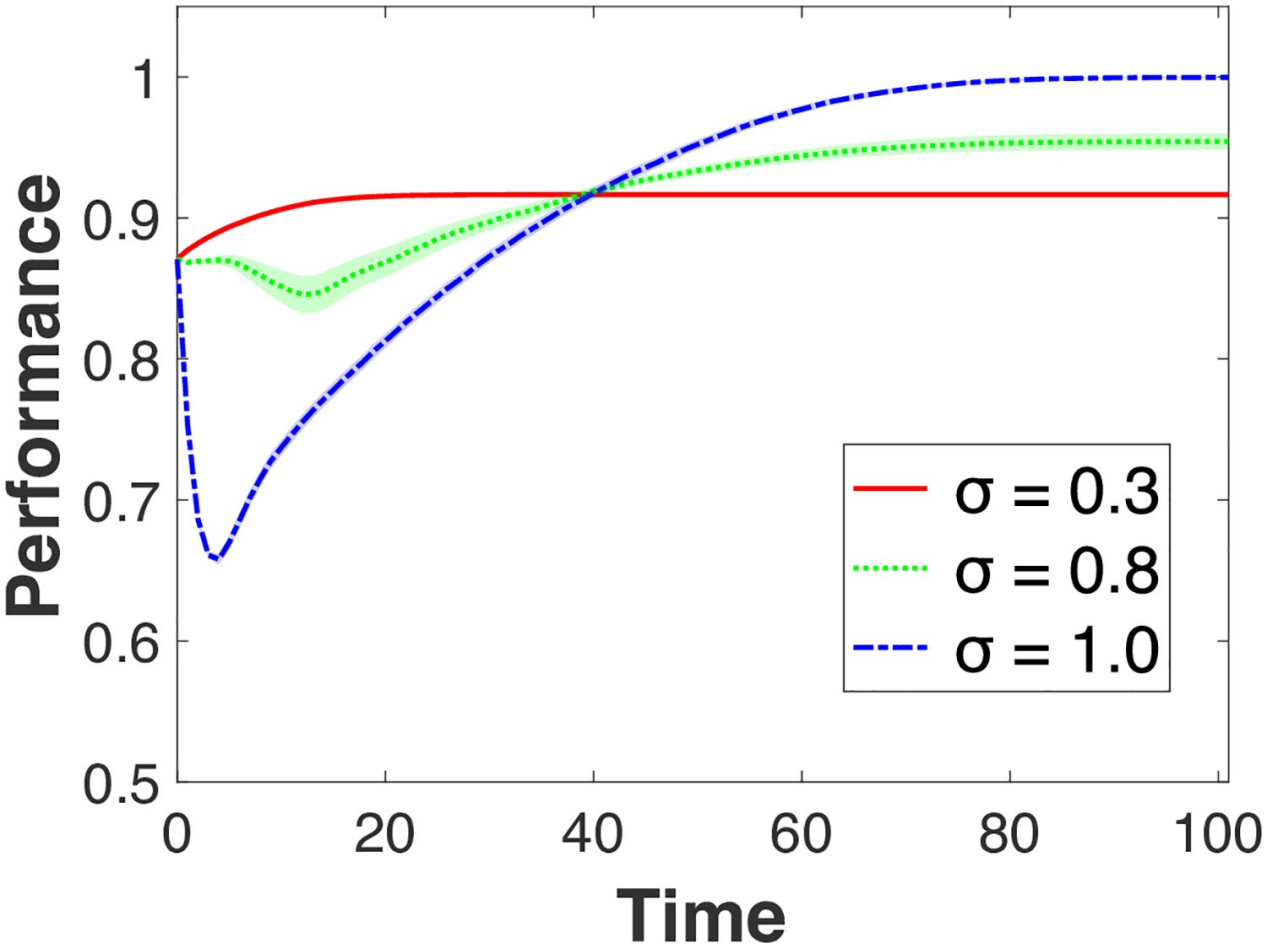
Effects under unequal peak case. The shades represent 95% confidence intervals.

So far, we have assumed that no reality is superior to the other. That is why the two peaks were of equal height. We now consider the case with two unequal peaks: one peak is higher than the other. To construct the case with unequal peaks, we increase performance levels on one side of the landscape by 20% (choosing a different percentage value yields similar results). Fig 7 reports the effect of search boundaries on performance for three levels of *σ*. The reported performance levels are normalized between 0 and 1.

Several things are noteworthy. First, as exploration boundary expands (i.e., a large *σ* range), the steady-state performance levels tend to be higher. When *σ* = 0.3 individuals do not search beyond the peak on which they are initially located, thus the resulting performance level is the average of the highest point of each peak. As *σ* increases, migration from one peak to the other takes place, and the higher peak tends to attract more individuals. Over time, everyone reaches the higher peak. Performance improves for increasing values of *σ* because the peaks have different heights and hence there is a superior solution in a region of the performance landscape. Under these conditions, engaging in broader exploration is beneficial.

### Probing the model assumptions

It is important to establish to what extent the previous results are an artifact of our modeling approach. Since our model explicitly considers the costs of variety-seeking, it might seem hardly surprising that we observe a performance decline. Yet, this is not an entirely obvious finding. To understand why, let’s consider the properties of a single-peak landscape compared to a multi-peak landscape. We created the landscapes over which the learning dynamic unfolds by following a modified version of March’s [3] model. We used *m*-dimensional binary vectors to represent points in the performance landscape. To create a single peak landscape, we randomly chose one of 2^*m*^ possible binary vectors. This chosen vector (called “reality”) is the location of the peak and its fitness is 1. Fitness levels for other vectors are the number of matching dimensions with the reality divided by *m*. For *k*-peak landscapes (*k*>1), we chose *k* binary vectors as realities such that Hamming distance between two adjacent realities is maximized. For example, when *k* = 2 and *m* = 6, we can choose (0,0,0,0,0,0) and (1,1,1,1,1,1) as two realities. When *k* = 3, we can choose a vector with three 0’s and three 1’s.

**Fig 8:**
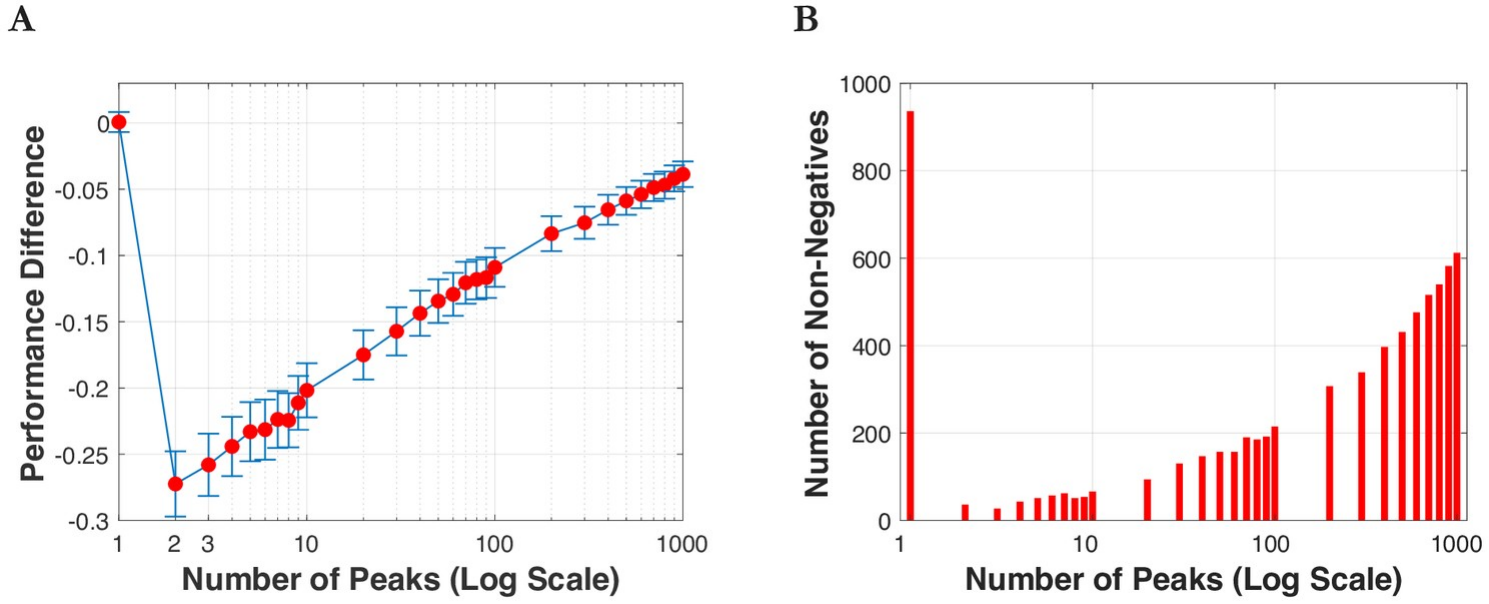
Number of peaks and learning consequences. **(A)** Performance difference after learning; negative values indicate a performance decline **(B)** Number of times the learning individual experiences non-negative performance changes out of 1,000 learning events.

Fig 8 plots the learning outcomes when one individual learns from another individual on landscapes that vary in the number of peaks. We considered two individuals on a landscape under two different scenarios: *μ* = 0.1 and 0.5. We present the case with *μ* = 0.1 only because we see a similar pattern for the other case. We placed two individuals on each side of the landscape. We let one individual learn from the other individual with a rate of *p* = 0.3. We created 1,000 learning events. For each event, we create two new individuals. For Fig 8A, we report the performance difference after learning with 95% confidence intervals. Negative values indicate a performance decline. For Fig 8B, we report the number of times the learning individual experiences non-negative performance changes. This captures the situation in which the learning individual lands on an equally good or better position after learning.

For the single-peak case, in 934 out of 1,000 times performance either increases or remains the same, while in only 66 times it drops slightly–which explains why ‘on average’ there is no performance decline. The results change dramatically in the presence of two or more peaks. When there are two peaks, on average performance drops by 27% compared to the initial performance (Fig 8A) and the learner experiences non-negative outcomes only 36 times out of 1,000 times (Fig 8B). In the case with 1,000 peaks, a ‘non-performance’ decline occurs more than 61% of the times (i.e., 612 out of 1,000 times) and when a performance decline is observed, the decline is very small–i.e., the performance difference is only 4% less than the initial performance. After the big drop in performance when there are two peaks, then performance gradually improves as the number of peaks increases. In sum, there is a significant difference between the single-peak and the multi-peak case. This explains why it is important to examine the variance hypothesis beyond a single-peak landscape and consider landscapes with two or more peaks. In the next paragraph, we generalize the previous results by re-running the simulation by creating a landscape with more than two peaks.

### Additional sensitivity analysis

To check whether our results are robust in other plausible scenarios, we conducted several sensitivity analyses. First, we checked whether our baseline results–the larger search boundaries are, the higher the initial costs–hold in the multi-peak case. To create a multi-peak landscape, we controlled the number of peaks on the landscape in the same way as we did in our two-peak case, and set the distance between one peak and the next constant: peaks would otherwise overlap with each other and, in the case with many peaks, the number of peaks would be smaller than the number we intend to have. The results show that the previous findings continue to hold in multi-peak landscapes. The initial costs that individuals experience as they learn from other individuals with diverse knowledge decrease as the number of peak increases. Intuitively, in a multi-peak landscape, an area of the landscape that was a ‘valley’ can be filled with new peaks. Because of these new peaks, individuals are more likely to land on a peak instead falling down a valley as they incorporate the knowledge of other individuals.

Furthermore, we examine the sensitivity of the results by varying the following parameters: (1) number of individuals (*n*); (2) number of dimensions in the external reality and individual knowledge (*m*); (3) learning rate (*p*) and the size of the learning target (*b*). We treat the parameter setting in Fig 2 as the baseline. First, we vary the number of individuals in the firm by setting this number to 50 and 150, respectively. Although not reported here, the main pattern of the results is almost identical to that of the baseline case in which the number of individuals is 100. While we believe this range of *n* is reasonable, decreasing or increasing *n* to an extreme level does not change the results. For example, an extremely small organization with two individuals would not reach the maximum performance level because there is not enough variety in knowledge. Yet, even in this case, the cost of variety-seeking would emerge if these two individuals occupy different peaks.

Next, we vary the number of dimensions in both the external reality and individual knowledge by setting them first to 10 and then to 100. Again, the pattern from the baseline results (when *m* = 50) is essentially the same. One noteworthy observation from this variation, however, is that performance begins to improve earlier and faster when the number of dimensions is low (e.g., *m* = 10). This is an intuitive result because individuals have a smaller number of knowledge dimensions to learn from the best performer. Then, we vary the learning rate (*p*) and the size of learning targets (*b*) by setting them at *p* = 0.1 and 0.9, and *b* = 1, 4, 50, and 99, respectively. The intuition from the case of varying *m* applies here as well. When the learning rate is high (e.g., *p* = 0.9), interpersonal learning improves performance faster but also experiences a smaller performance decline initially. Likewise, increasing the size of the learning target improves performance because it raises the likelihood of learning from the best performer and, at the same time, reduces the likelihood of incorporating the ‘wrong’ knowledge.

We also consider the case in which an individual has the ability to (imperfectly) forecast the learning outcome (i.e., foresight). The screening function in Knudsen and Levinthal [35, p. 41] allows for this ability by setting the probability of accepting the new alternative as a function of the difference between the status quo and this new alternative. We experimented with various levels of screening ability from zero (no foresight) to close to infinity (near-perfect screening or foresight). In our baseline case, the acceptance probability is set equal to 1 (very low foresight), regardless of the fitness difference. We find that, unless the screening ability gets closer to the near-perfect foresight condition, the basic pattern of our results still holds. To summarize, these additional analyses (which are available from the authors upon request) suggest that the main results are robust to parameter variations. Finally, we relax the assumption that individuals are able to identify the better performer out of *b* learning targets by allowing for errors in perceiving other individuals’ performances. We do so by adding a randomly drawn number from a normal distribution in which the mean is equal to zero and the variance equal to 1 in one case, and 9 in the other. The transition from two-peak to one-peak convergence takes place near *σ* = 0.8 when the variance is 1, and *σ* = 0.78 when the variance is 9. Although not reported here, the overall pattern still holds.

## Discussion and conclusions

Following the publication of March’s [3] paper, the terms exploration and exploitation have become almost ubiquitous, dominating “organizational analyses of technological innovation, organizational design, organizational adaptation, organizational learning, competitive advantage, and organizational survival” [20, pp. 693]. On the premise that a firm’s long-term success depends on its ability to balance exploitation and exploration, a large body of theoretical and empirical research has examined this fundamental tension in organizational learning and adaptation [4]. Indeed, one of the main challenges for a firm’s long-term performance is to conduct sufficient exploitation to ensure its current viability, while devoting enough energy to exploration to ensure its future viability. As Levinthal and March [36, pp. 107] noted, attaining an optimal mix of exploration and exploitation is difficult because of “the tendency of rapid learners and successful organizations to reduce the resources allocated to exploration.” A central tenet of research on learning is that variety in knowledge over time improves organizational learning and performance. Preserving and expanding the requisite variety are the ultimate goals of exploration. Yet this research provides no precise guidelines about how broad exploration should be.

Building on this basic insight, prior research has examined how managers can leverage the organizational structure to foster boundary spanning interactions [2, 32, 37]. Connecting otherwise isolated individuals or groups allows superior solutions to be identified and exploited–an insight that is consistent with early organizational theorists’ intuition that creating lateral relations (from direct contacts to task forces to more complex integrating roles) across separate organizational units [38] enhances a firm’s decision-making and long-term performance. A few scholars have also argued that “cultivating a broad external search has an opportunity cost as it takes attention away from other activities internal to the firm” [1, pp. 280]. This point resonates with attention-based theories that emphasize how any allocation of attention implies focusing on some opportunities while foregoing other (possibly even better) opportunities [39, 40]. From this perspective, exposure to diverse sources of knowledge provides access to the requisite variety, but also increases the costs of efforts at variety-seeking. Drawing from these two streams of research, we examined the tension underlying the variance hypothesis. We developed a simulation model that allows us to probe the conditions under which variety-seeking enhances or hinders firm performance.

The results of the simulation depict a more nuanced picture than those offered in previous computational studies that model search in terms of interpersonal learning. In particular, we show that there are situations in which the benefits from variety-seeking might accrue to individuals engaging in local as opposed to more distant exploration. In the model, this is captured by individuals’ initial location on the performance landscape. When individuals are located close to the top of a given peak, the variety needed to reach the top may be available locally rather than in a more distant location of performance landscape. In this type of learning environment, interacting with individuals located on different peaks might prove of little help to climb the focal peak–even as individuals incorporate the knowledge of better performing others located on those peaks.

This explains why expanding the search breadth may entail diminishing returns [9, 41]. As Dahlander et al. [1] found out in their study of the search behaviors of élite boundary spanners at IBM, low external search breadth–coupled with high attention to localized information sources–is an effective search approach for generating innovative outcomes; besides, it does not entail the costs of a long-jump. Focused exploration, in other words, might prove a viable search strategy. For instance, Corning took advantage of the broad knowledge base it had accumulated in specialty glass when, in 1966, the British Post Office approached it asking for a glass that could be used to make optical glass fibers for long-distance telecommunication applications–which Corning ultimately did by leveraging its long-standing expertise in fused silica [42]. Since the range of possible applications for a firm’s knowledge base is typically wider than its applications at any given moment, firms can capitalize on previous technological investments by transferring over time knowledge already available in-house [43–45]. The case of Gorilla Glass discussed earlier illustrates this process as well.

A very different learning environment is the one in which performance-enhancing knowledge is spread out over the performance landscape. In this case, a firm can increase the likelihood of gaining access to the requisite variety by expanding the breadth of its search activities. The results of the simulation afford a more nuanced window into the conditions under which variety-seeking is a viable strategy. In particular, they reveal how the type of learning environment determines the point beyond which variety-seeking (exploration) translates into a sharp performance decline. The presence of this critical point explains why departing from familiar learning paths is difficult and, when it happens, a performance decline is to be expected. The logic of this explanation resonates with the finding that adaptive search through exploration may be costly, especially when it entails “breaking off neighborhood search before exhausting the potential of local improvements” [46, pp. 94]. The existence of a curvilinear–inverted U-shape–relationship between search breadth and performance is in line with prior empirical research showing that having a number of external linkages with different types of partners (e.g., consumers, suppliers, universities, competitor, and so on) increases “the likelihood of innovation not only by directly increasing the flow of useful external knowledge, but also by increasing the chances of productive complementarities between external and internal knowledge” [47, p. 1704]. Despite the benefits of collaborating with different types of partners, Love, Roper, and Vahter [47] further argue that there are limits to the value of external linkages because search is costly. These costs may arise from the need to “write appropriate contractual agreements for numerous formal linkages, and to maintain these linkages through time,” or the cognitive limits of management in processing information “since the span of attention of any individual is limited” [47, pp. 1704]. Regardless of whether learning unfolds within or across organizational boundaries, our model suggests that the point at which the costs of variety-seeking outweigh the benefits depends on the type of learning environment. Differences in transition patterns reflect the extent to which individuals’ knowledge is more or less specialized. As knowledge becomes more specialized, individuals have to broaden their search to find better-performing others whose knowledge can be recombined with their own to improve performance, which explains why the transition point occurs for higher levels of search breadth. However, after this point, the costs of broadening search–i.e., the costs of variety-seeking–tend to increase even more, thus hampering performance.

An important question that deserves more careful consideration is whether, in the process of exploring new solutions, the transition point preceding a sharp performance decline can be detected. Where this critical point lies might be a function of a firm’s prior history, identity, or accumulated knowledge. When the exploration of alternative courses of action implies departing from what a firm does, the costs of this move may drastically reduce or even offset the corresponding benefits. One of the main challenges for managers is to recognize when exploration means incorporating knowledge that is hardly compatible with a firm’s existing knowledge. The issue of how expansive or localized exploration should be, therefore, demands further investigation. By modeling different learning environments, where individual knowledge exhibits different levels of specialization, our study represents one of the first attempts to address this important question more systematically.

### Limits and future research directions

There are several extensions of our model that future research may find worth pursuing. We identified the nature of the learning environment as a critical factor in shaping the relationship between variety-seeking and performance. However, other contingent factors might be important as well. Experimental research has shown, for instance, that groups whose members are involved in stable collaborations tend to develop transactive memory systems or shared mental models that help them coordinate their actions more effectively and perform better than groups lacking such stable collaborations [48–52]. Developing shared mental models is an essential aspect of learning in groups [50] as it fosters the diffusion and recombination of knowledge, and a more accurate sense of ‘who knows what’ inside the group [21, 29, 53–55]. In our model, we do not examine the role of transactive memory systems. Over time, however, individuals will end up sharing the same or very similar understandings as they interact and incorporate each other’s knowledge. Making this link more explicit by allowing individuals to remember ‘who knows what’ from previous interactions is an interesting extension of our model: the existence of transactive memory systems could help individuals recognize which knowledge should be incorporated and which one ignored, therefore reducing the risk of experiencing a performance decline.

Our approach to modeling interpersonal learning is consistent with a well-established tradition in computational studies of learning in organizations. Although there are obvious limitations to this approach, the purpose of a model is to generate insight that has some empirical plausibility–even if it does not capture more specific details of real-world organizations. For instance, in larger organizations the learning dynamic–as we model it–is better suited to capturing interpersonal learning at the team level: as organizations grow larger, individuals are unlikely to interact with everyone else–and especially so with those further up in the hierarchy. In relatively smaller organizations such as a start-up or in organizations with a flat organizational structure, however, our model retains its empirical plausibility. As discussed above, our interpersonal learning model captures important aspects of the learning dynamic unfolding in firms like Pixar, whose organizational structure is relatively flat and there are close interactions not only within and between team members, but also between them and senior managers. At Pixar, for example, when a movie director and his or her team feel in need of assistance, they can call upon a group of senior directors–known as the *brain trust*–and show them the current version of the work in progress. After the meeting, “it’s up to the director of the movie and his or her team to decide what to do with the advice; there are no mandatory notes, and the brain trust has no authority” [15, p. 6]. The interaction between the team working on a movie and the brain trust members has proven critical in fostering learning and making a movie better.

When variety-seeking involves learning across organizational boundaries, the experience accumulated in processing information from a variety of different sources contributes to reducing the costs of broadening search. There is indeed a “learning process involved in managing external innovation relationships, so that previous experiences shape the relationship between current breadth of linkages and innovation outputs” [47, pp. 1704]. Similarly, intra-organizational learning can be enhanced when organizational routines and practices are in place that facilitate the exchange of knowledge and the reconciliation of differences that do not necessarily reflect the specificity of the local reality. For instance, Four Seasons has established a task force, composed of experienced managers and staff, that “helps establish norms and helps people understand how Four Season does things” [16, pp. 9], in addition to assisting the local managers in getting the new property up and running. By contributing to instilling the same core knowledge in the management of all properties, the task force has been instrumental in fostering the exchange of knowledge between different locations, as well as helping managers realize when knowledge is more or less specialized, and so less easily transferable.

In the analysis, we kept the external reality stable–yet, in the sensitivity analyses, we changed the complexity of the performance landscape by varying dimensions in the external reality and individual knowledge. Several studies have explicitly modeled the interplay between learning within organizations and environmental dynamism, and how it affects efforts to resolve the tension between exploration and exploitation [33]. For instance, exploration becomes even more critical, and organizations should consider allocating more resources to variety-seeking, when the level of environmental turbulence increases. Yet, even in this case, the extent to which exploration should be more or less focused remains an open question. Examining interpersonal learning under varying degrees of environmental dynamism is a natural extension of our model. These represent avenues for future research that we hope will contribute to a better understanding of the complexities of learning and performance in organizations.

## Supporting information

S1 Appendix Results under the NK model setting. (DOCX)
