# Supplementary material for: Variety-seeking, learning and performance
Source: PLoS One. 2021 Mar 8;16(3):e0247034. doi: 10.1371/journal.pone.0247034 (PMC7939370; doi:10.1371/journal.pone.0247034)
Supplement: S1 Appendix — (DOCX) [file pone.0247034.s001.docx]

**S1 Appendix.** Results under the NK model setting

In this Appendix, we run the same learning model as in Figure 2 on NK landscapes. The main idea we want to demonstrate is that the variance hypothesis is generally supported under NK landscapes regardless of the level of K (i.e., degree of ruggedness). Thus, the NK model is not designed for examining the cost of variety seeking. To create NK-based performance landscapes, we set N=10 and varied the level of K. Figure A1 reports the results.

| 1. K = 1 | 1. K = 3 |
| --- | --- |
|  |  |
| **(c)** K = 5 | **(d)** K = 7 |
|  |  |

**Figure A1.** Interpersonal Learning on NK Landscapes

In all the figures with low to high levels of K, the performance patterns over time do not show signs of performance decline even in the case in which individuals do not have any foresight about the landscape. What is notable is that, as K increases, broad exploration (high *σ*) tends to improve slower than do searches with focused exploration (low *σ*). But there is no performance decline. We observed the same pattern with *μ* = 0.5 where individuals are more scattered in the beginning. Also note that for large K’s (e.g., K=7), we started the value for *σ* from 0.4 because with a lower value of *σ*, sometimes no other individual knowledge is located within the search boundary. This requires too much computing time to complete the search process.
